# Nitric oxide decreases intestinal haemorrhagic lesions in rat anaphylaxis independently of mast cell activation

**DOI:** 10.1080/09629359791893

**Published:** 1997-02

**Authors:** J. Carvalho Tavares, A. Moreno, M. Sánchez Crespo

**Affiliations:** 1 Instituto de Biología y Geneética Molecular CSIC-Facultad de Medicina Valladolid 47005 Spain

## Abstract

The purpose of this study is to assess the role of nitric oxide (NO) in the intestinal lesions of passive anaphylaxis, since this experimental model resembles necrotizing enterocolitis. Sprague-Dawley rats were sensitized with IgE anti-dinitrophenol monoclonal antibody. Extravasation of protein-rich plasma and haemorrhagia were measured in the small intestine. Plasma histamine was measured to assess mast cell activation. The effect of exogenous NO on the lesions was assessed by using two structurally unrelated NO-donors: sodium nitroprusside and S-nitroso-Nacetyl-penicillamine (SNAP). An increased basal production of NO was observed in cells taken after anaphylaxis, associated with a reduced response to platelet-activating factor, interleukin 1beta, and IgE/DNP-bovine serum albumin complexes. The response to bacterial lipopolysaccharide and dibutyryl cyclic adenosine monophosphate (AMP) was enhanced 24 h after challenge, but at earlier times was not significantly different from that observed in controls. Treatment with either sodium nitroprusside or SNAP produced a significant reduction of the haemorrhagic lesions, which are a hallmark of rat anaphylaxis. The extravasation of protein-rich plasma was not influenced by NO-donors. The increase of plasma histamine elicited by the anaphylactic challenge was not influenced by SNAP treatment. NO-donors protect intestinal haemorrhagic lesions of rat anaphylaxis by a mechanism apparently independent of mast cell histamine release.


Mediators of Inflammation, 6, 25–31 (1997)

### Nitric oxide decreases intestinal haemorrhagic lesions in rat anaphylaxis independently of mast cell activation

J. Carvalho Tavares, A. Moreno and M. Sánchez Crespo[CA]

Instituto de Biología y Genética Molecular, CSIC-Facultad de Medicina, 47005-Valladolid, Spain

[CA]Corresponding Author
Tel: (+34) 83-423273
Fax: (+34) 83-423588
Email: mscres@wamba.cpd.uva.es

**THE purpose of this study is to assess the role of nitric oxide (NO) in the intestinal lesions of passive anaphylaxis, since this experimental model resembles necrotizing enterocolitis. Sprague-Dawley rats were sensitized with IgE anti-dinitrophenol monoclonal antibody. Extravasation of protein-rich plasma and haemorrhagia were measured in the small intestine. Plasma histamine was measured to assess mast cell activation. The effect of exogenous NO on the lesions was assessed by using two structurally unrelated NO-donors: sodium nitroprusside and S-nitroso-N-acetyl-penicillamine (SNAP). An increased basal production of NO was observed in cells taken after anaphylaxis, associated with a reduced response to platelet-activating factor, interleukin 1β, and IgE/DNP-bovine serum albumin complexes. The response to bacterial lipopolysaccharide and dibutyryl cyclic adenosine monophosphate (AMP) was enhanced 24 h after challenge, but at earlier times was not significantly different from that observed in controls. Treatment with either sodium nitroprusside or SNAP produced a significant reduction of the haemorrhagic lesions, which are a hallmark of rat anaphylaxis. The extravasation of protein-rich plasma was not influenced by NO-donors. The increase of plasma histamine elicited by the anaphylactic challenge was not influenced by SNAP treatment. NO-donors protect intestinal haemorrhagic lesions of rat anaphylaxis by a mechanism apparently independent of mast cell histamine release.**

**Key words:** CD23, Endotoxin, Inflammation, Necrotizing enterocolitis, Platelet-activating factor

### Introduction

Necrosis of the small intestine similar to that produced by endotoxin, tumour necrosis factor[1] or platelet-activating factor[2] is a feature characteristic of rat anaphylaxis,[3,4] its pathogenetic mechanism being a direct consequence of triggering the cascade of chemical mediators from mast cells by IgE-dependent mechanisms. This necrosis is histologically identical to that seen in neonatal necrotizing enterocolitis. In a previous study we have shown that platelet-activating factor (PAF) is a major effector of this response. Interestingly, the haemorrhagic lesions were dependent on different chemical mediators than those involved in the production of protein-rich plasma extravasation.[5] Nitric oxide (NO) plays an important role in cell signalling (for review see Ref. 6) and it has been found in a number of cells that are either directly or indirectly activated in anaphylactic reactions. In addition, NO mediates inflammatory responses and defence mechanisms including killing of microorganisms, oedema and apoptosis. The analysis of the actual role of NO in immunoinflammatory reactions is a matter of debate since it has been reported to produce either tissue injury[7–9] or attenuation of the damage induced by other agents.[10–12]

NO affects the function of immune cells by different mechanisms, e.g. interaction with guanylyl cyclase or interference with the transcription factor NF-kB, which among many other functions is the main regulator of the molecules that are involved in the production of endothelial cell activation.[13,14] The effect of NO as a mediator of gastrointestinal mucosal defence is currently associated with its action as an endogenous scavenger of various free radical species, and there is convincing evidence that

© 1997 Rapid Science Publishers administration of large amounts of NO does not cause detectable damage to the mucosa or vasculature of the intestine.[15]

Since there is a host of signals that may lead to the production of NO, analysis of the mechanism involved in NO production during anaphylactic reactions should consider a number of alternatives. Thus, release of NO by rat serosal mast cells has been reported.[16] Macrophages are another source of NO and a number of autacoids released from mast cells could potentially lead to the induction of NO production by macrophages, e.g. PAF[17] and tumour necrosis factor-alpha (TNF-α).[18] Moreover, it has been recently reported that IgE-dependent activation of macrophages via FcεRII/CD23 (low affinity receptor for the Fc portion of IgE) leads to a strong induction of NO production in both rat[19] and human macrophages[20] that allows killing of parasites. In keeping with these data, induction of the inducible isoform of NO synthase during anaphylaxis might occur via either direct or indirect IgE-dependent mechanisms. Even though NO is produced during anaphylactic challenge, its main role in the process may be difficult to understand in view of both its pleiotropic effects and different time-frames in which it is generated. In this study we have addressed: (1) the effect of IgE-dependent anaphylaxis on the generation of NO by rat peritoneal macrophages; (2) the effect of NO-generating compounds on the necrotic lesions of the small intestine produced in IgE-dependent reactions; (3) the effect of NO donors on histamine release in IgE-dependent anaphylaxis.

### Materials and Methods

### Materials and drugs

Monoclonal anti-dinitrophenol (DNP) IgE was obtained from a secreting hybridoma[21] grown as ascites tumours in BALB/c mice and purified by ammonium sulphate precipitation as described.[4] The amount of specific antibody was calculated from classical precipitation reactions using antigen and several dilutions of the antibody solution.[22] Dinitrophenol-bovine serum albumin (BSA) containing 8 mol of DNP per mol of BSA was prepared according to the method of Eisen.[23] IgE-DNP-BSA immune complexes at equivalence were made by overnight incubation at 4°C of antigen and the antibody solution followed by extensive washing.[22] 1-Hexadecyl-2-acetyl-*sn*-glycero-3-phosphocholine (PAF), 4β-phorbol 12β-myristate 13α-acetate (PMA), S-nitroso-N-acetyl-penicillamine (SNAP), N-acetyl-DL-penicillamine, and $N^G$-methyl-arginine (L-NMA) were from Sigma Chemical Company, St Louis, MO. Sodium nitroprusside (SNP) was from Fides Laboratories, Spain. TNF-α and interleukin 1β were from Genzyme Corp., Cambridge, MA.

### In vivo experimental design

Male Sprague-Dawley rats of about 200 g body weight were passively sensitized with IgE monoclonal antibody (i.p., in 0.2 ml of a phosphate-buffered isotonic saline solution, pH 7.4) at the dose of 12 mg protein/kg measured by the method of Bradford.[24] Anaphylactic challenge was performed by i.v. injection with 0.7 mg/kg DNP-BSA together with 20 mg/kg Evans blue dye (EB) in phosphate-buffered isotonic saline solution to assay protein-rich plasma extravasation (see below) 18 h after sensitization.[25] The experimental protocol was approved by our Institutional Review Board.

### Assay of NO production by peritoneal macrophages ex vivo

Peritoneal cells were collected in 50 ml of Dulbecco's modified Eagle medium (DMEM) without phenol red, pelleted and resuspended in the same medium supplemented with 100 U/ml penicillin, 100 μg/ml streptomycin, 50 μg/ml gentamicin, 0.5 mM L-arginine, 2 mM glutamine and 10% heat-inactivated fetal calf serum. Non-adherent cells were removed by washing after 2 h of incubation, and the production of NO was assessed after incubation at 37°C in an atmosphere containing 5% $CO_2$. More than 95% of the adherent cells were macrophages as assessed by both their morphological appearance and functional ability to engulf non-opsonized zymosan particles. Mast cells were $< 0.5\%$ of total adherent cells as judged from staining with toluidine blue solution at pH 3.5.

### Determination of NO and nitrite

NO released from macrophage cultures was determined by the accumulation of nitrite.[26] The cell cultures ($5 \times 10^5$ in 1 ml of phenol red-free medium) were filled with 100 μl of a solution of 1 mM sulphanilic acid and 100 mM HCl (final concentration). After incubation for 5 min the medium was aspirated and centrifuged in an Eppendorf microcentrifuge. Fifty μl of naphthylenediamine (1 mM in the assay) were added to the samples, and the reaction was completed after 15 min of incubation. The absorbance at 548 nm was compared with a standard of $NaNO_2$, and the production of NO was expressed as nmol of $NO_2^-$/mg protein.

### Evaluation of protein-rich plasma extravasation

Vascular permeability changes were evaluated by the EB extravasation method, as described by Jancar *et al.*[27] The EB was injected into the jugular vein together with the antigen. The jejunum-ileum was dissected, weighed, and put in formamide (4 ml/g wet tissue at 20°C for 24 h) to extract the EB. The concentration of EB was determined by spectrophotometry at a wavelength of 620 nm. The results were plotted on a standard curve of EB, and the content of each sample was expressed as μg per gram of dry weight tissue. Dry weight was obtained by weighing the tissue after 24 h at 60°C.

### Extraction and quantitation of haemoglobin from intestinal tissue

The concentration of haemoglobin was determined colorimetrically by the cyanomethaemoglobin method[28] using reagents from Sigma, according to the modifications carried out by Tavares de Lima *et al.*[29] Briefly, fragments of the ileal-jejunal portions of the small intestine were excised and minced in 2 ml of potassium cyanide and hexacyanoferrate solution. After 24 h at room temperature in the dark, the tissue was removed, the sample was centrifuged and the optical density of the supernatant was determined spectrophotometrically at 546 nm. The concentration of haemoglobin was calculated by comparison with a standard curve and was expressed as mg per gram of dry weight tissue. There is no significant interference of EB in this colorimetric assay, making both assays in samples from the same animal possible.

### Assay of histamine released after antigen challenge

Histamine was measured in plasma by a modification of the method of Shore *et al.*[30] Briefly, blood was taken from a femoral artery cannulated with a polyethylene catheter, anticoagulated with edetic acid (EDTA), cooled at 4°C and centrifuged to remove cells. Histamine was extracted from 200 μl plasma samples by 0.4 N perchloric acid treatment, followed by sequential extractions in butanol and heptane. The fluorometric assay was carried out after condensation of histamine with *o*-phthalaldehyde at alkaline pH. The fluorescent product was measured at room temperature with wavelength excitation at 360 nm and emission at 450 nm. The concentration of histamine was determined from a standard curve constructed with known concentrations of histamine. For these experiments, the animals were anaesthetized with pentobarbital sodium (60 mg/kg body weight), and a tracheostomy was then performed to facilitate breathing.

### Statistical analyses

Data are expressed as the mean ± SEM. For comparison of two groups of samples normally distributed, the Student's two-tailed *t*-test was used to analyse differences for significance. For comparison of two groups of samples not normally distributed, the Mann-Whitney *U*-test was used. Statistical procedures were performed using a data base and statistical package (Instat, GraphPAD Software Inc., San Diego, CA), $P < 0.05$ was considered significant.

### Results

### Anaphylactic challenge triggers NO production by peritoneal adherent cells and modifies their response to agonists

Adherent cells collected after induction of anaphylaxis spontaneously produced increased amounts of nitrite compared with cells from animals treated with either antigen or antibody alone (Fig. 1). This production was suppressed by the specific NO synthase inhibitor L-NMA, but not by D-NMA suggesting the involvement of the L-arginine pathway in the production of NO under these conditions. The time elapsed after anaphylaxis influenced the ability of adherent peritoneal cells to produce NO, since maximal production was observed in cells collected 24 h after anaphylaxis (Fig. 1). Further approaches to analyse the mechanism of the enhanced production of NO was carried out by examining the response of adherent peritoneal cells to a set of agonists that are either released after anaphylactic challenge or act through well-known signalling pathways. As shown in Fig. 2, the pattern of response to these agonists presented significant differences that can be summarized as follows. PMA was the only agonist that elicited an enhanced nitrite production in cells collected 10 min after anaphylaxis compared with controls. The response to IL-1β, IgE/DNP complexes and PAF was reduced compared with controls, except in cells taken 24 h after challenge. LPS and dibutyryl cyclic adenosine monophosphate (AMP) elicited on cells

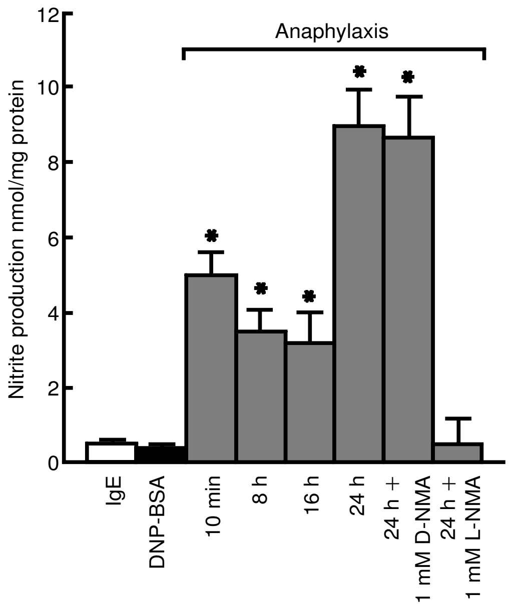

FIG. 1. Spontaneous production of nitrite by adherent peritoneal cells in culture after anaphylactic challenge. Peritoneal cells were collected at the times indicated after anaphylactic challenge by washing the peritoneum with 50 ml of DMEM, followed by centrifugation and resuspension in 10 ml of the same medium. Cells non-adherent to plastic dishes were removed after 2 h and the production of nitrate/nitrite assayed 24 h thereafter with the Griess reagents. Control animals included rats treated with either antibody or antigen alone and collected 24 h thereafter. The cross-hatched columns show the accumulation of nitrite in the presence of either the NO synthase inhibitor L-NMA or its isomer D-NMA. Data represent mean ± SEM of 12 to 15 animals in each group. $^{*}P < 0.05$.

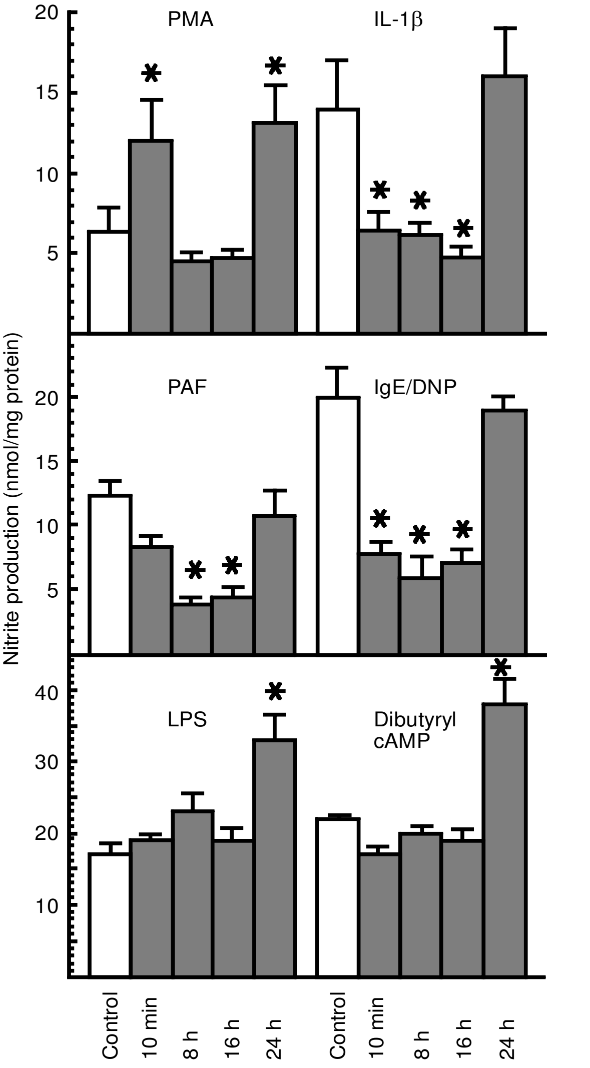

FIG. 2. Production of nitrite by adherent peritoneal cells from rats undergoing anaphylactic challenge stimulated with different agonists. Cells were taken after anaphylaxis as explained in the legend to Fig. 1. Adherent cells were incubated as indicated with 32 nM PMA, 1 nM IL-1β, 20 nM PAF, 100 μg/ml IgE/DNP-BSA equivalence preformed insoluble immune complexes, 10 μg/ml LPS and 0.5 mM dibutyryl cyclic AMP. The production of nitrite was assayed 24 h after addition of stimuli. Data represent mean ± SEM from 12 to 15 animals. $^{*}P < 0.05$ compared with cells from control animals sensitized with i.p. antibody and challenged with vehicle.

from DNP-challenged animals a response similar to that observed in control rats, except when the cells were collected 24 hours after anaphylaxis, on which they elicited an increased production of nitrate. TNF-α at concentrations up to 1 nM did not induce significant NO production.

### Treatment with SNP and SNAP prior to the anaphylactic reaction decreases haemorrhagic necrosis of the small intestine

Attempts to delineate the effect of NO on rat anaphylaxis were performed with SNP and SNAP, two NO-generating compounds that are not structurally related. Treatment with SNP at the dose of 0.1 mg/kg, i.p., prior to anaphylactic challenge, induced a significant reduction of the haemorrhagic necrosis without affecting extravasation significantly as judged from the accumulation of EB (Fig. 3). A similar effect was observed with SNAP at the dose of 1 mg/kg i.p. prior to the challenge with DNP-BSA. A group of rats treated with 1.4 mg/kg of N-acetyl-penicillamine showed no protection at all, which indicates that the effect of SNAP should be attributed to its nitrosyl moiety. Inhibition of NO synthesis with L-NMA at the dose of 10 mg/kg did not elicit significant changes on the haemorrhagic necrosis, suggesting that the amount of NO that can be produced early after anaphylactic challenge from the constitutive isoform of NO synthase does not attain a suitable concentration to prevent injury.

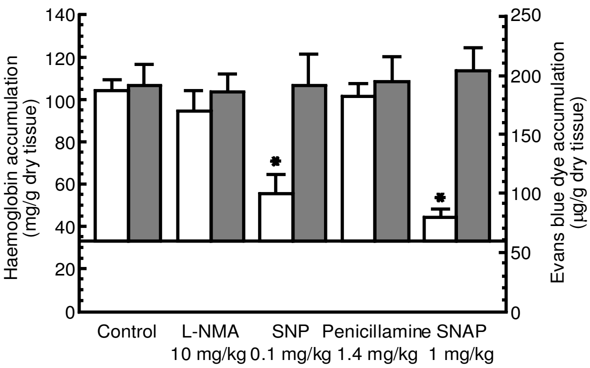

FIG. 3. Effect of NO-generating compounds on the accumulation of both Evans blue dye and haemoglobin in the small intestine of IgE-sensitized rats undergoing anaphylactic challenge. Drugs were administered 10 min prior to antigen challenge at the doses indicated. Rats were sacrificed 15 min after anaphylaxis to obtain small intestine samples. The line at the base of the columns shows mean control values obtained in 10 IgE-sensitized rats challenged with vehicle. Open columns indicate haemoglobin accumulation. Hatched bars indicate Evans blue dye accumulation. Data represent mean ± SEM from 14 to 16 rats. $*P < 0.05$.

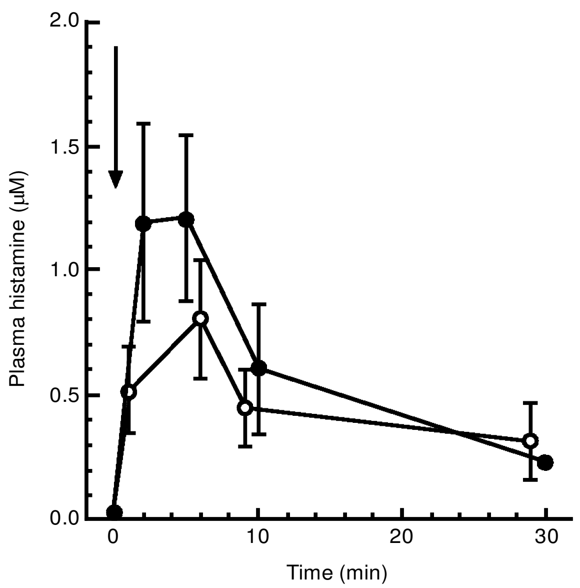

FIG. 4. Plasma histamine concentration after anaphylactic challenge. The femoral artery of rats was cannulated with polyethylene catheter and 0.4 ml blood samples were taken before (arrow) and at different times after DNP-BSA administration. Open circles indicate rats treated with vehicle. Closed circles indicate rats pretreated with 1 mg/kg SNAP. Data represent mean ± SEM of 15–18 animals at each point. Differences were not significant at any of the times tested.

### Treatment with SNAP does not affect the levels of plasma histamine after anaphylactic challenge

In order to obtain some information about the level at which the protective effect of SNAP on the anaphylactic reaction was exerted, histamine plasma levels were assayed at different times after DNP-BSA challenge as an indicator of mast cell activation and degranulation. Injection of DNP-BSA to sensitized rats produced a rapid increase of plasma histamine levels from 0.028 ± 0.007 µM to 0.8 ± 0.24 µM 5 min after challenge. This was followed by a decline of plasma histamine after 10 min. Assay of histamine plasma levels in rats pretreated with SNAP at both the usual dose of 1 mg/kg (Fig. 4) and at 4 mg/kg (not shown) did not induce any significant decrease of plasma histamine levels.

### Discussion

In previous studies we have observed that triggering of anaphylactic reactions in rats passively sensitized with monoclonal antibody produces shock, extravasation of protein rich plasma, and severe lesions of the small intestine characterized by coagulative necrosis of the epithelial layer, oedema in the *lamina propria*, and extravasation of blood red cells into the interstitium.[4,5] These changes are due to the triggering of the cascade of chemical mediators released from mast cells, and are analogous to those produced by the infusion of PAF.[2,31] In the present study we show that triggering of the anaphylactic reaction also induces NO production by adherent peritoneal cells and modifies the response of these cells to NO-inducers. This seems to be due to the sudden release of a variety of autacoids that initiate chemical signalling. Analysis of the different molecules that could be implicated in this signalling should include products derived from mast cells as well as direct activation of macrophages by IgE-antigen complexes via the low affinity FcεRII/CD23 receptor. Activation of NO production through this receptor seems to be of potential importance since it is involved in parasite killings.[20] Among the list of mast-cell derived products that could induce NO production on macrophages we focused on PAF, IL-1β, and TNF-α, based on their well-documented ability to induce NO production.[18,32,33] On the other hand, to address the chance of a direct triggering of macrophages via CD23 antigen we have utilized preformed IgE-DNP complexes. In addition, we selected other agonists in view of their unique properties of inducing NO synthase by well-known biochemical pathways. Thus, LPS was used as a good reference compound that is currently used to induce NO production. Dibutyryl cyclic AMP was selected in view of its well-known effect on mesangial cells, macrophages and smooth muscle vascular cells by a mechanism different from that involved in NO induction by IL-1β.[34] PMA was used as an inductor of NO production by protein kinase C-dependent mechanisms.[35] Analysis of the responses to these agonists after anaphylaxis showed down-regulation of the responses to some of them, namely IL-1β, PAF and IgE/DNP complexes, suggesting that cells could have reacted with these stimuli prior to *ex vivo* stimulation, this event leading to a cross-talk of signals. This is in keeping with what could be expected on the basis of the autacoids that are released after IgE challenge, and could explain the up-regulation of NO production observed in cells removed after anaphylaxis. In keeping with this interpretation, an increased concentration of NO has been detected in the exhaled air of patients after allergen-induced late asthmatic reactions,[36] and this has been considered a good indicator of the degree of inflammation of the airways. This also agrees with the enhanced production of NO by peritoneal cells taken after the induction of immune-complex peritonitis.[17]

To define the pathophysiological consequences of the modulation of NO production during anaphylaxis we first carried out experiments with L-NMA to inhibit NO production. This treatment did not modify the extent of both haemorrhagia and extravasation in animals undergoing anaphylactic challenge. This is different from the reported increase of epithelial permeability produced by inhibition of NO synthase via activation of mucosal mast cells, which has been associated with the release of PAF, histamine and superoxide.[37] A likely explanation could be the time-frame in which these changes occur, since in that report they were maximal 30 min after treatment with the NO synthase inhibitor. Another reason could be that NO synthase inhibition on its own initiates the generation of mediators by mast cells and, thereby, enhances the release of chemical mediators triggered by anaphylaxis. Since the magnitude of tissue injury is very prominent in our model, it seems difficult to enhance damage by pharmacological procedures.

On the other hand, treatment with two structurally unrelated NO-generating compounds showed a significant protection of the haemorrhagic component of the lesions without reducing protein-rich plasma extravasation. It is not fully unexpected that haemorrhagia and exudation show different pharmacological modulation. A likely explanation for this finding is that the occurrence of haemorrhagia might require a wide disruption of the integrity of endothelial cells, compared with the mild changes needed for protein-rich plasma extravasation to occur. This agrees with the results we observed antagonizing the PAF receptor.[5]

Since a portion of the effect of NO on gastrointestinal mucosal defence has been associated to its role as an endogenous modulator of mast cell reactivity,[16,38] we measured plasma histamine after anaphylactic challenge as a reporter of mast cell activation. Plasma histamine concentrations in SNAP-treated animals were not significantly different from those measured in non-treated animals after antigen challenge, making it unlikely that the alleviation of intestinal haemorrhagia produced by NO-generating compounds could only be explained by an overall inhibition of mast cell activation.[39] In fact, the assay of plasma histamine is only an indicator of mast cell activation, whereas other mediators, e.g. PAF, seem to be the actual effectors of the injury.[5] However, the recent report of the blockade of PAF-induced bowel injury by NO-donors[40] strongly suggests that these compounds operate downstream the mast cell activation step.

Potential targets of NO are redox-sensitive signalling pathways which include protein tyrosine phosphatases and kinases that are affected by covalent interactions with sulphydryl groups.[41,42] This seems of interest because protein tyrosine phosphorylation reactions are involved in biochemical signalling in the microcirculation.[43] NO also has effects on the transcription factor NF-kB. This action is linked to the ability of NO to scavenge and inactivate superoxide anion,[44] and might be of central importance in vascular biology because NF-kB is involved in endothelial cell activation by promoting the expression of adhesion molecules and proinflammatory cytokines.[13,14] Transcriptional activation of the p50 subunit of NF-kB has been demonstrated in the small bowel of mice treated with either PAF or TNF-α at concentrations lower than those required to produce systemic changes.[45] Therefore, the effect of NO-generating compounds in the attenuation of the haemorrhagic necrosis associated with IgE-dependent mast cell activation should be related to these types of interactions rather than to the inhibition of the release of mediators from mast cells.

### References

1. Alonso A, Carvalho J, Alonso-Torre SR, Núñez L, Boscá L, Sánchez Crespo M. Nitric oxide synthesis in rat peritoneal macrophages is induced by IgE/DNP complexes and cyclic AMP analogues. Evidence in favor of a common signaling mechanism. *J Immunol* 1995; **154**: 6475–6483.
2. González-Crussi F, Hsueh W. Experimental model of ischemic bowel necrosis: the role of platelet-activating factor and endotoxin. *Am J Pathol* 1983; **112**: 127–135.
3. Fell BF, Boyne R, Cuthbertson DP. Intestinal lesions following histamine liberation in the rat. *J Pathol Bact* 1961; **82**: 445–452.

4. Fernández-Gallardo S, Gijón MA, García C, Furió V, Liu FT, Sánchez Crespo M. The role of PAF and peptidoleukotrienes in the vascular disturbances of rat passive anaphylaxis. *Br J Pharmacol* 1992; **105**: 119–125.
5. Pellon MI, Steil AA, Furio V, Sánchez Crespo M. Study of the effector mechanism involved in the production of hemorrhagic necrosis of the small intestine in rat passive anaphylaxis. *Br J Pharmacol* 1994; **112**: 1101–1108.
6. Moncada S, Palmer RMJ, Higgs EA. Nitric oxide: physiology, pathophysiology, and pharmacology. *Pharmacol Reviews* 1991; **43**: 109–141.
7. Mulligan MS, Moncada S, Ward PA. Protective effects of inhibitors of nitric oxide synthase in immune complex-induced vasculitis. *Br J Pharmacol* 1992; **197**: 1159–1162.
8. Palmer RMJ, Bridge LN, Foxwell A, Moncada S. The role of nitric oxide in endothelial cell damage and its inhibition by glucocorticoids. *Br J Pharmacol* 1992; **105**: 11–12.
9. Miller MJS, Grisham MB. Nitric oxide as a mediator of inflammation? You had better believe it. *Mediators of Inflammation* 1995; **4**: 387–396.
10. Boughton-Smith NK, Hutcheson I, Deakin AM, Whittle BJR, Moncada S. Protective effect of S-nitroso-N-acetyl-penicillamine in endotoxin-induced acute intestinal damage in the rat. *Eur J Pharmacol* 1990; **191**: 485–488.
11. Kubes P, Suzuki M, Granger DN. Nitric oxide: an endogenous modulator of leukocyte adhesion. *Proc Natl Acad Sci USA* 1991; **88**: 4651–4655.
12. Kubes P, Wallace JL. Nitric oxide as a mediator of gastrointestinal mucosal injury?—Say it ain't so. *Mediators of Inflammation* 1995; **4**: 397–405.
13. De Caterina R, Libbi P, Peng HB, Thannickal VJ, Rajavashisth TB, Gimbrone MA, Shin WS, Liao JK. Nitric oxide decreases cytokine-induced activation. *J Clin Invest* 1995; **96**: 60–68.
14. Ferran C, Millam MT, Csizmadia V, Cooper JT, Brostjan C, Bach FH, Winkler H. Inhibition of NF-kB by pyrrolidine dithiocarbamate blocks endothelial cell activation. *Biochem Biophys Res Commun* 1995; **214**: 212–223.
15. Kubes P, Reinhardt P, Payne D, Woodman RC. Excess nitric oxide does not cause cellular, vascular or mucosal dysfunction in the cat small intestine. *Am J Physiol* 1995; **269**: G34–G41.
16. Salvemini D, Masini E, Anggard E, Mannaioni F, Vane J. Synthesis of nitric oxide-like factor from L-arginine by rat serosal mast cells: stimulation of guanylate cyclase and inhibition of platelet aggregation. *Biochem Biophys Res Commun* 1990; **169**: 596–601.
17. Steil AA, García MC, Alonso A, Sánchez Crespo M, Boscá L. Platelet-activating factor is the effector of protein-rich plasma extravasation and nitric oxide synthase induction in rat immune complex peritonitis. *Br J Pharmacol* 1995; **114**: 895–901.
18. Gordon JR, Galli SJ. Mast cells as a source of both preformed and immunologically inducible TNF-α/cachectin. *Nature* 1990; **346**: 274–276.
19. Tracey KJ, Beutler B, Lowry SJ, Merryweather J, Wolpe S, Milsark IW, Hariri RJ, Fahey III TJ, Zentella A, Albert J, Shires GT, Cerami A. Shock and tissue injury induced by recombinant human cachectin. *Science* 1986; **234**: 470–475.
20. Vouldoukis I, Riveros-Moreno V, Dugas B, Ouazz F, Becherel P, Debre P, Moncada S, Mossalayi MD. The killing of Leishmania major by human macrophages is mediated by nitric oxide induced after ligation of the FcεRII/CD23 surface antigen. *Proc Natl Acad Sci USA* 1995; **92**: 7804–7808.
21. Liu FT, Bohn JW, Ferry EL, Yamamoto H, Molinaro CA, Sherman LA, Klinkman N, Katz DJ. Monoclonal dinitrophenyl-specific murine IgE antibody: preparation, isolation and characterization. *J Immunol* 1980; **124**: 2728–2734.
22. Hudson L, Hay FC. Antibody interactions with antigen. In: Hudson L, Hay FC, ed. *Practical Immunology*. Oxford: Blackwell Scientific Publications, 1996; 88–93.
23. Eisen HN. Preparation of purified anti-2, 4-dinitrophenyl antibodies. In: Eisen HN, ed. *Methods in Medical Research. Vol. 10*. Chicago: Yearbook Medical Publishers, 1964; 94–120.
24. Bradford M. A rapid and sensitive method for the quantitation of microgram quantities of protein using the principle of protein-dye binding. *Anal Biochem* 1976; **72**: 248–254.
25. Leng W, Kuo CG, Qureshi R, Jackchik BA. Role of leukotrienes in vascular changes in the rat mesentery and skin in anaphylaxis. *J Immunol* 1988; **140**: 2361–2368.
26. Green LC, Wagner DA, Glogowski J, Skipper PL, Wishnok JS, Tannenbaum SR. Analysis of nitrate, nitrite, and [$^{15}$N]nitrate in biological fluids. *Anal Biochem* 1982; **126**: 131–138.
27. Jancar S, Sirois MG, Carrier J, Braquet P, Sirois P. PAF induces rat plasma extravasation and releases eicosanoids during anaphylaxis. *Inflammation* 1991; **15**: 347–354.
28. Van Kampen EJ, Ziljtra WG. Standardization of hemoglobinometry. II. The hemoglobincyanide method. *Clin Chim Acta* 1961; **6**: 358–551.
29. Tavares de Lima W, Sirois P, Jancar S. Immune-complex alveolitis in the rat: evidence for platelet-activating factor and leukotrienes as mediators of the vascular lesions. *Eur J Pharmacol* 1992; **213**: 63–70.
30. Shore PA, Burkhalter A, Cohn VH Jr, Pieroni L, Guilloson JJ, Debre P, Arock P. A method for the fluorometric assay of histamine in tissues. *J Pharmacol* 1959; **127**: 182–194.
31. Sánchez Crespo M, Alonso F, Iñarrea P, Alvarez V, Egido J. Vascular actions of synthetic paf-acether (a synthetic platelet-activating factor) in the rat: evidence for a platelet-independent mechanism. *Immunopharmacology* 1982; **4**: 173–185.
32. Galli SJ. New concepts about the mast cell. *New Engl J Med* 1993; **328**: 257–265.
33. Hogaboam CM, Befus AD, Wallace JL. Modulation of rat mast cell reactivity by IL-1β. Divergent effects on nitric oxide and platelet-activating factor release. *J Immunol* 1993; **151**: 3767–3774.
34. Kunz D, Mühl H, Walker G, Pfeischifter J. Two distinct signaling pathways trigger the expression of nitric oxide synthase in rat renal mesangial cells. *Proc Natl Acad Sci USA* 1994; **91**: 5387–5391,
35. Hortelano S, Genaro AM, Boscá L. Phorbol esters induce nitric oxide synthase activity in rat hepatocytes. Antagonism with the induction elicited by lipopolysaccharide. *J Biol Chem* 1992; **267**: 24937–24940.
36. Kharitonov SA, O'Connor BJ, Evans DJ, Barnes PJ. Allergen-induced late asthmatic reactions are associated with elevation of exhaled nitric oxide. *Am J Resp Crit Med* 1995; **151**: 1894–1899.
37. Kanwar S, Wallace JL, Befus D, Kubes P. Nitric oxide synthesis inhibition increases epithelial permeability via mast cells. *Am J Physiol* 1994; **266**: G222–G229.
38. Bidri M, Becherel PA, Le Goff L, *et al.* Involvement of cyclic nucleotides in the immunomodulatory effects of nitric oxide on murine mast cells. *Biochem Biophys Res Commun* 1995; **210**: 507–517.
39. Kubes P, Kanwar S, Niu XF, Gaboury JP. Nitric oxide synthesis inhibition induces leukocyte adhesion via superoxide and mast cells. *FASEB J* 1993; **7**: 1293–1299.
40. MacKendrick W, Caplan M, Hsueh W. Endogenous nitric oxide protects against platelet-activating factor-induced bowel injury in the rat. *Pediatr Res* 1993; **34**: 807–812.
41. Lander HM, Sehajpal PK, Novogrodsky A. Nitric oxide signaling: a possible role for G proteins. *J Immunol* 1993; **151**: 7182–7187.
42. Stamler JS. Redox signaling: nitrosylation and related target interactions of nitric oxide. *Cell* 1994; **78**: 931–936.
43. Kim D, Durán W. Platelet-activating factor stimulates protein tyrosine kinase in the hamster cheek pouch microcirculation. *Am J Physiol* 1994; **268**: H399–H403.
44. Schreck R, Rieber RP, Baeuerle PA. Reactive oxygen intermediates as widely used messengers in the activation of the NF-kB transcription factor and HIV-1. *EMBO J* 1991; **10**: 2247–2258.
45. Tan X, Sun X, Gonzalez-Crussi FX, Gonzalez-Crussi F, Hsue W. PAF and TNF increase the precursor of NF-kappa B p50 mRNA in mouse intestine: quantitative analysis by competitive PCR. *Biochim Biophys Acta* 1994; **1215**: 157–162.

ACKNOWLEDGEMENTS. This paper has been supported by grants from Dirección General de Investigación Científica y Técnica (DGICYT grant no.: PM92-0006), and Fondo de Investigación Sanitaria (FIS grant no.: 95/1765). J.C.T. is the recipient of a grant from Ministerio Español de Asuntos Exteriores (Programa Mutis).

**Received 27 September 1996; accepted 20 October 1996**
